# A Guide to Left Bundle Branch Area Pacing Using Stylet-Driven Pacing Leads

**DOI:** 10.3389/fcvm.2022.844152

**Published:** 2022-02-21

**Authors:** Jan De Pooter, Aurelien Wauters, Frederic Van Heuverswyn, Jean-Benoit Le polain de Waroux

**Affiliations:** ^1^Heart Center, University Hospital Ghent, Ghent, Belgium; ^2^Service de Cardiologie, Clinique Saint Pierre, Ottignies, Belgium; ^3^Department of Cardiology, AZ Sint Jan, Bruges, Belgium

**Keywords:** left bundle branch area pacing, stylet-driven pacing leads, lumen-less pacing lead, conduction system pacing, stylet-driven extendable screw lead

## Abstract

Left bundle branch area pacing (LBBAP) has emerged as a novel pacing modality which aims to capture the left bundle branch area and avoids the detrimental effects of right ventricular pacing. Current approaches for LBBAP have been developed using lumen-less pacing leads (LLL). Expanding the tools and leads for LBBAP might contribute to a wider adoption of this technique. Standard stylet-driven pacing leads (SDL) differ from current LLL as they are characterized by a wider lead body diameter, are stylet-supported and often have a non-isodiametric extendable helix design. Although LBBAP can be performed safely with SDL, the implant technique of LBBAP differs compared to LLL. In the current overview we describe in detail how different types of SDL can be used to target a deep septal position and provide a practical guide on how to achieve LBBAP using SDL.

## Introduction

Conduction system pacing (CSP) aims to pace the ventricles by capturing the conduction system at either the level of the His bundle (His bundle pacing, HBP) or the left bundle branch (left bundle branch area pacing, LBBAP). These new pacing techniques were developed to avoid the detrimental effects of pacing induced dyssynchrony with right ventricular pacing by offering more physiologic activation of the heart (1–3). Of the two techniques, HBP is deemed the most physiological as it captures the ventricular conduction system at its proximal origin, but its clinical applicability is limited by high pacing thresholds, low sensing amplitudes, oversensing issues, and a greater number of lead revisions (4, 5). Left bundle branch area pacing has subsequently emerged as an attractive alternative as it provides comparable physiological pacing to HBP but with lower pacing thresholds, higher sensing amplitudes, and more stable lead positions (3, 6–9). To obtain LBBAP, the pacing lead is positioned deep into the ventricular septum, along the course of the left bundle branch. Until now, LBBAP has been performed almost exclusively with a lumen-less pacing lead (LLL) with a fixed helix design (7–9). Detailed operator guides on how to perform LBBAP using LLL (LLL-LBBAP) have been previously published (10). Recently, LBBAP using standard stylet-driven pacing leads (SDL) has been reported to be safe and feasible (11, 12). However, due to the differences in lead and helix design, LBBAP with SDL (SDL-LBBAP) requires different handling and lead preparation. In the current overview, we describe in detail how LBBAP can be safely performed with different types of SDL and highlight the relevant differences with respect to LLL-LBBAP.

## Stylet- and Sheath-Guided Conduction System Pacing

Early in the evolution of CSP, HBP was attempted with SDL and custom-curved stylets (13). Although HBP was feasible with this approach, implant success was low and pacing thresholds remained often high and unstable. In 2006, Zanon et al. described a new approach for HBP using a long preshaped delivery sheath to guide the pacing lead toward the His bundle area (14). The use of such delivery sheaths allowed for a more stable position and better contact of the pacing lead with the His bundle area. It also allowed the use of a narrow-caliber LLL, which rapidly appeared associated with better long-term results and lead stability. With this sheath-guided approach, implant success of HBP increased to 90%. Since then, sheath-guided HBP has become the standard approach and is now also used to achieve LBBAP (9, 10). The widespread adoption of the sheath-guided method for LBBAP has also been driven by the use of LLL which requires, due to absence of stylet support, a dedicated delivery sheath to be directed toward the septum.

Current guiding sheaths for LBBAP are similar to those used for HBP. Several delivery sheaths are commercially available for CSP (both HBP and LBBAP) with the majority having a double curved design (Supplementary Figure 1A). The wide primary curve allows to cross the tricuspid valve toward the interventricular septum while the smaller secondary curve ensures lead positioning perpendicular to the septum. Currently available guiding sheaths differ with respect to size and angulation of the curves and have been developed to address differences in cardiac size or to target different sites of the conduction system. Deflectable-single curve-sheaths are also proposed for CSP but appears less appropriate for LBBAP as they have a tendency to bring the pacing lead in a potentially dangerous anterior position. Details regarding different delivery sheaths have been previously published (15).

## Lumen-Less vs. Stylet-Driven Pacing Leads: Differences in Lead Design

Different pacing leads used for LBBAP, are shown in Supplementary Figure 1B with details on lead and helix design summarized in Table 1. Largest experience with LBBAP has been obtained with a single type of LLL (SelectSecure, 3830 pacing lead, Medtronic Inc., Minneapolis, USA). Due to the absence of an inner lumen, the lead body measures only 4.1 Fr and the fixed helix (1.8 mm length) design results in an isodiametric lead. The electrically active helix of the SelectSecure pacing lead facilitates conduction system capture in both unipolar and bipolar pacing mode.

**Table 1:** Lead specifications of different stylet-driven and lumen-less pacing leads used for left bundle branch area pacing.

**Lead name**	**SelectSecure 3830**	**Solia S**	**Ingevity**	**Tendril 2088TC**
Manufacturer	Medtronic	Biotronik	Boston Scientific	Abbott
Lead design	Lumen-less	Stylet-driven	Stylet-driven	Stylet-driven
Lead length (cm)	59/69/74	45/53/60	45/52/59	46/52/58/65/85/100
Lead body diameter (mm/Fr)	1.4 (4.1)	1.8 (5.6)	1.9 (5.7)	1.9 (5.8)
Helix design	Fixed, non-retractable	Retractable	Retractable	Retractable
Cathode design (Lead tip electrode)	Electrical active helix	Electrical active helix	Electrical active helix	Electrical active helix
Tip electrode length (mm)	1.8	1.8	1.8	2.0
Tip electrode surface area (mm^2^)	3.6	4.5	4.5	6.9
Tip to ring electrode spacing (mm)	9	10	10.7	10
Anode ring electrode surface area (mm^2^)	16.9	17.4	20	16
Anode ring electrode width (mm)	Not specified	1.9 (5.9)	2.0 (6.0)	Not specified
Outer isolation	Polyurethane	Polyurethane/Silicone	Polyurethane (55D)	Optim^TM^
Inner isolation	Silicone/ETE	Silicone	Silicone	Silicone
Rotations to extend helix				
With straight stylet	NA	5–10	7	6–11
With J- stylet	NA	5–10	8	9–14
Maximal number of rotations	NA	17 (45 cm length) 21 (53 cm length) 23 (60 cm length)	30	No maximum specified
Steroid eluting	Yes	Yes	Yes	Yes
Steroid eluting	Beclomethasone Diproprionate	Dexamethasone Acetate	Dexacomethasone Acetate	Dexamethasone Sodium Phosphate

Standard stylet-driven pacing leads differ from LLL with respect to several important features. Standard stylet-driven pacing leads have an inner lumen which allows for stylet insertion. As a result, the lead body of SDL is wider than LLL and usually measures >5.5 Fr. Standard stylet-driven pacing leads are also stiffer than LLL when the stylet is inserted. The SDL helix has an extendable-retractable design. Fully extended the SDL helix measures 1.8–2.0 mm in length, similar as the SelectSecure lead, but due to a wider diameter, the electrically active helix surface of SDL is larger compared to LLL. The lead body of SDL consists of an inner and outer coil which are separated by silicon insulation and rotate independently from each other. The inner coil is connected distally to the helix and proximally to the rotating pin of the pacing lead. Clockwise rotation of the connector pin allows extending the helix. However, when rotating the outer lead body of SDL, care must be taken to ensure that rotations of the outer lead body are adequately transferred to the inner coil. If the inner and outer coils do not rotate simultaneously, retraction of extended helices might occur and will hamper lead advancement in the septum.

## LBBAP Implant Technique Using Lumen-Less Pacing Leads

Lumen-less pacing leads–left bundle branch area pacing has been described in detail by Huang et al. and most implantation techniques represent small variations on Huangs approach (10). With this approach, a single type of LLL (SelectSecure, model 3830, Medtronic Inc., Minneapolis, USA) is used and dedicated delivery sheaths are mandatory with this type of lead as it lacks the support of a stylet. Two sheaths, with a fixed double curve (C315 His, Medtronic Inc., Minneapolis, USA) or a deflectable curve (C304 and C304 His, Medtronic Inc., Minneapolis, USA) are available for use. The delivery sheath is advanced to the right ventricle over a J-tip guidewire. Using a right anterior oblique (RAO 20–30°) view on fluoroscopy can help to avoid unwanted CS cannulation, which tends to happen frequently due to the double curvature of the sheaths. Once in the right ventricle, the pacing lead is advanced through the delivery sheath and both sheath and lead are retracted to target the upper part of the septum. The implant height on the septum is determined by localizing the His bundle region and targeting a septal position >1 cm from the His region toward the apex in a RAO view. Slight counter clockwise rotation of the sheath and lead combination allows for perpendicular positioning on the septum which is best confirmed, in our experience, in a 25–30° left anterior oblique fluoroscopic (LAO) view. A small amount of contrast may also be injected to delineate the right septal border and confirm the appropriate septal position. In this position, unipolar pace mapping at the lead tip typically reveals a wide “W” shaped QRS morphology in lead V1 of the 12-lead surface electrocardiogram (ECG). The SelectSecure pacing lead is screwed into the septum by clockwise rotation of the outer lead body with the delivery sheath in close contact with the right septum in order to maintain a stable lead position on the septum. Lead advancement into the septum is further guided by unipolar pacing impedance, contrast injection, assessment of paced QRS morphology, observation of fixation beats, or presence of a left bundle branch potential on the unipolar lead tip electrogram (10, 16, 17). As the pacing lead reaches the course of the left bundle branch, the paced “W” shaped QRS morphology in lead V1 gradually changes to a narrow QRS morphology with a terminal r-wave in lead V1 (so-called incomplete right bundle branch block morphology). As such 12-lead ECG monitoring is mandatory for successful LBBAP. Different criteria to confirm capture of the left bundle branch and differentiate left bundle branch pacing from left sided myocardial capture have been proposed, although currently no consensus exists (10, 16, 18, 19).

## LBBAP Implant Technique Using SDL

With SDL-LBBAP, delivery sheath manipulation and septal positioning is similar to that of LLL-LBBAP. Given the length of SDL helix, extending the helix alone will not penetrate the septum deep enough to achieve LBBAP. Therefore, similar to LLL-LBBAP, rotating the outer lead body of SDL is mandatory to access deep septal position (11, 12). However, when rotating the outer lead body of SDL, retraction of the helix may occur due to fixation in the tissue causing the outer coil to turn over the inner coil. Below we provide details on our approach to achieve LBBAP with different types of SDL.

### Solia S Pacing Lead, Biotronik

The Solia S pacing lead (Biotronik, SE & Co., KG, Germany) is a 5.6 Fr SDL with an extendable helix design. The lead body consists of an outer and inner coil, with the latter connected to the electrical active helix. When used for LBBAP, our approach is to extend the helix in advance, generally before septal positioning is attempted (Figure 1.1A). Alternately, one can map the His area and perform pace mapping with the helix withdrawn, which give less chance to snag the tricuspid valvular apparatus. However, when performing pace mapping on the right side of the septum, unipolar impedances might be more accurate with extended helix and might allow a better reference impedance when screwing into the septum. To avoid helix retraction when clockwise rotating the outer lead body, the inner coil of Solia S lead needs to be pretensioned before insertion. Therefore, the green stylet-guide is connected to the lead pin and pressed against the silicone coating at the proximal portion of the lead (Figure 1.1B). To build up the torque on the inner coil, this green stylet-guide tool is rotated 8–10 times clockwise (Figure 1.1C). As such, the inner coil builds up tension and rotations of the outer lead body are better transferred in a one-to-one relation to the inner coil and helix. This preparation step avoids unwanted helix retraction during screwing. To maintain the tension, the green stylet-guide is kept on the pin of the pacing lead while screwing the Solia S lead toward a deep septal position. Advancement of the Solia S lead into the septum is further facilitated by fast rotation (to overcome resistance at the right septal subendocardial layer) and by keeping the stylet advanced to the tip of the pacing lead. The stylet and stylet guide are kept in position until the final position is reached (Figure 1.1D).

**Figure 1:**
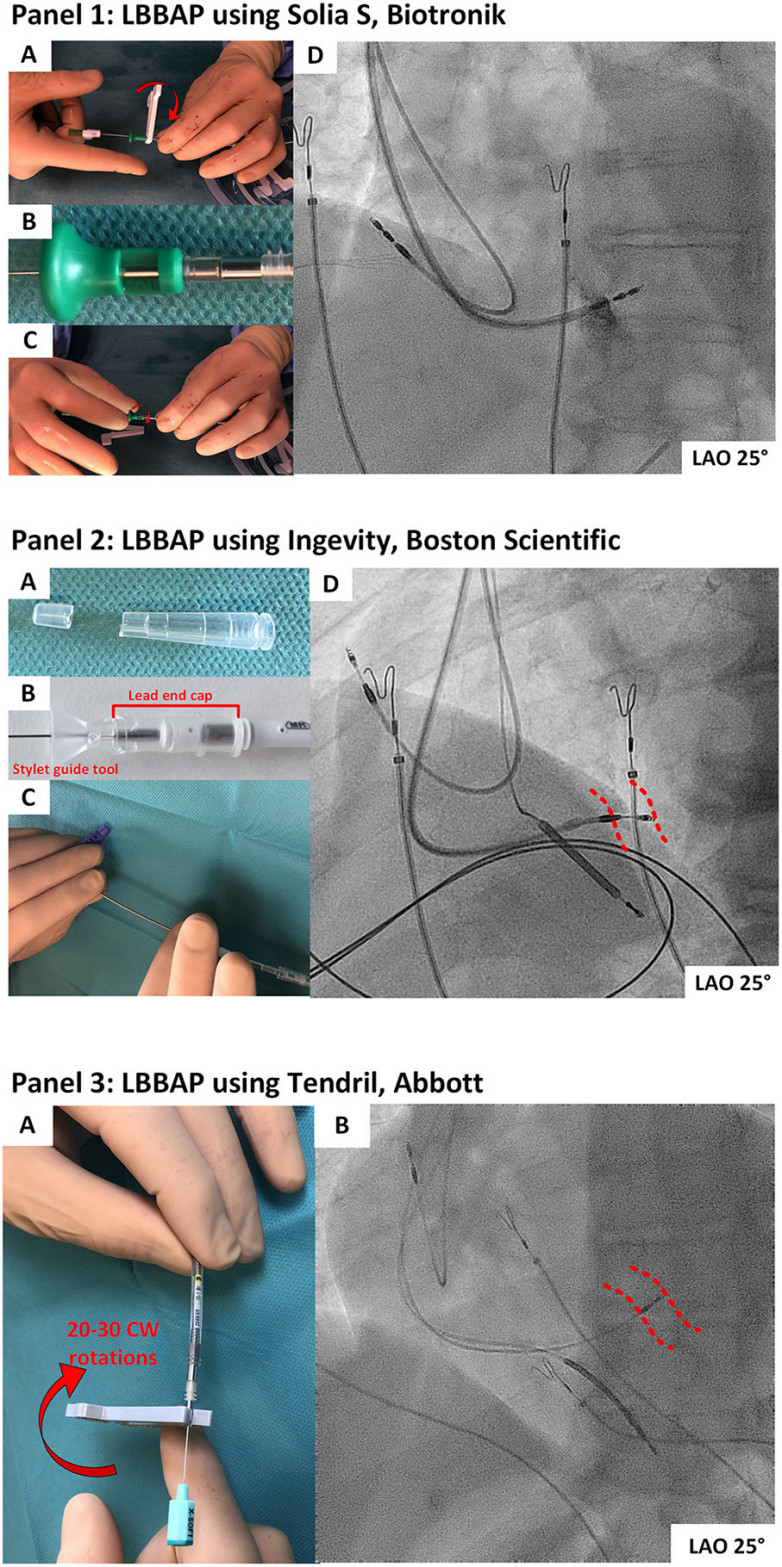
**(1.1)** LBBAP using the Solia S lead (Biotronik). **(1A)** The helix is extended using the standard clip-on-tool with 10–15 clockwise rotations. **(1B)** The green stylet insertion tool is connected to the pin of the pacing lead and the silicon rubber separating the inner and outer coil. **(1C)** Tension to the lead is applied with 10 additional clockwise rotations on the green stylet insertion tool. **(1D)** Deep septal position of the Solia-S lead on fluoroscopy. **(1.2)** LBBAP using the Ingevity pacing lead (Boston Scientific). **(2A)** The closed end of a regular lead cap is cut. **(2B)** The lead cap is advanced over the lead pin toward the silicone rubber at the proximal part of the lead. The stylet insertion tool is forced between the lead end cap and the pin of the pacing lead and pushed toward the proximal lead part. **(2C)** The Ingevity lead is screwed in a deep septal position by applying clockwise rotations on the outer lead body. **(2D)** Deep septal position of the Ingevity lead on fluoroscopy. The red dotted lines indicate the septal borders. **(1.3)** LBBAP using the Tendril 2088TC pacing lead (Abbott). **(3A)** The tendril pacing lead can be screwed toward a deep septal position by continuous rotations with the clip-on tool on the pin of the pacing lead. As the helix grips into the tissue, it will further advance into the septum and pull the lead body toward a deep septal position. Often 20–30 clockwise (CW) rotations are needed. **(3B)** Deep septal position of a Tendril pacing lead. The red dotted lines indicate the septal borders.

### Ingevity Lead, Boston Scientific

The Ingevity pacing lead (Boston Scientific Inc., Marlborough, MA, USA) is a 5.7 Fr diameter SDL with an extendable helix design. The electrically active helix is extended in advance using the standard clip-on-tool. To screw the Ingevity lead in a deep septal position two approaches are used. With the first approach, clockwise rotations on the outer lead body are applied. This generally leads to helix retraction, as described before, and further advancement in the septum becomes hampered, as the helix is no longer exposed. Helix retraction is often suggested by a sudden increase in pacing impedance (sometimes up to >2,000 Ohms). The helix needs to be extended once again using the clip-on-tool. Afterwards, new clockwise rotations on the outer lead body can be applied to further advance the lead. These maneuvers are repeated until a deep septal position is reached. A second method consists in extending the helix in advance and fixating the helix and inner coil to the outer lead coil. As the stylet-guide tool of this lead does not get over the proximal silicone seal of the lead, the tension created on the inner coil is not maintained. However, a custom-made fixation tool can be made from an IS-1 lead-end cap (20). First, the tip of an IS-1 lead end-cap is cut-off and the opened lead end-cap is slided over the proximal end of the pacing lead (beyond the proximal electrode). Secondly, with the stylet fully inserted in the lead, the stylet-guide tool is advanced onto the connector pin and around 15 rotations of the stylet-guide tool are applied to expose the helix and pretension the inner coil. Finally, without releasing the built-up tension, the lead-end cap is pulled back until the insertion tool is forced between the lead pin and the lead end cap. This technique allows to maintain the pretension on the inner coil and avoid helix retraction when clockwise rotations of the outer lead body of the Ingevity are applied (Figure 1.2A–D). With both approaches the stylet remains advanced to the tip of the pacing lead while screwing as this facilitates lead advancement into the septum.

### Tendril 2088TC, Abbott

The Tendril 2088TC lead (Abbott, Inc., USA) is a 5.8 Fr SDL with an extended helix measuring 2 mm in length. The outer isolation of this lead consists of a polymer (Optim^TM^) made of silicone and polyurethane. This particular insulation has the potential to become damaged when subject to rotations applied on the outer lead body. Therefore, rotating the outer lead body of the Tendril is not recommended to obtain a deep septal position. However, the helix extension mechanism of this lead is protected from overturning and helix fracture has not been described, even with numerous rotations. With the helix extended, the Tendril pacing lead is positioned at the right side of the septum and unipolar pace mapping is performed. The Tendril pacing lead is advanced into the septal tissue by continuous clockwise rotation of the connector pin using the standard clip-on-tool delivered with the lead (Figure 1.3A,B). As the helix grips the septal tissue, continuous rotation of the lead pin will advance the helix and lead body further into the septum. The tapered transition between the helix and lead body facilitates the advancement of the Tendril lead in the septum. Often, 20–30 rotations on the lead pin are needed before the lead reaches the left side of the septum.

## Precautions and Potential Pitfalls When Using SDL for LBBAP

With the stylet inserted, SDL are stiffer than LLL and care must be taken not to perforate through the septum when implanting LBBAP leads. It is recommended to screw SDL in deep septal positions under fluoroscopic guidance and with continuous monitoring of the unipolar impedance and paced QRS. The lead implant depth can be assessed with contrast injection and based on the fluoroscopic landmarks of the pacing electrodes and interelectrode distance (Table 1). As the lead advances into the septum, the unipolar pacing impedance tends to rise initially but decrease by 50–100 ohms as it approaches the left sided septal border. If the impedance drops by more than 200 ohms during screwing, further lead advancement is not recommended as this indicates that the helix is at the edge of the left sided septum. Absolute values of unipolar pacing impedance depend on the type, length, and design of the SDL. As the Boston Scientific Ingevity lead is developed as a high impedance pacing lead, it demonstrates higher unipolar impedances than the Tendril or the Solia S leads. Therefore, we recommend using unipolar pacing impedance at the right side of the septum as an individual reference for impedance monitoring during screwing. Furthermore, unipolar pacing impedances measured from the stylet are reported to be comparable to unipolar pacing impedances measured from the lead pin (21).

A further potential pitfall of SDL is the risk of entanglement of the exposed helix in the right-sided subendocardial tissue. This so-called entanglement effect has previously been described in a cadaver model with LLL targeted to a deep septal position but can occur with any type of pacing lead (22). Entanglement of the helix occurs when the helix does not get grip on the septal tissue but instead becomes trapped in the septal subendocardial tissue. Prolonged rotation of the lead body without lead advancement into the septum, may eventually result in complete helix entrapment and difficulty in repositioning the lead (23). If entanglement is suspected, counter clockwise rotation and slight traction on the lead body while maintaining tension on the lead, usually untangles the lead.

## Discussion: The use of SDL in LBBAP and Future Perspectives

Reported experience with LBBAP using SDL is limited (11, 12). In a recent study, SDL- and LLL-LBBAP yielded similar implant success rates, procedural safety and pacing characteristics (11). Although larger studies are needed to confirm these results, SDL may offer advantages for LBBAP for several reasons. The thicker lead body of SDL together with the support of the stylet results in excellent torquability and stiffness when targeting deep septal positions with rotations applied on the outer lead body effectively transferred to the distal part of the lead and helix. Compared to LLL, unwanted twisting of the lead at the entry of the delivery sheath during implant is rarely observed. Furthermore, the larger lead body diameter of SDL also allows for an improved tissue grip when rotating the lead body. As such, and despite the larger lead body diameter, SDL are characterized by an easy penetration into the septum. Additionally, unipolar lead impedances can be monitored directly on the stylet of SDL, rather than through connection of the crocodile clamps on the lead pin (21). This approach avoids repetitive connection and disconnection of the clamps, limits less lead body rotations, and offers continuous unipolar pacing and reliable impedance monitoring during screwing. Another advantage with stylet-driven leads is that in the unfortunate event of post-operative lead dislodgment, the lead may be repositioned in a “conventional” right ventricular position without having to regain venous access. Disadvantages include the requirement for lead preparation and particular precautions to avoid unwinding of the helix during deep septal lead positioning for SDL leads with extendable helices. An additional drawback is that His bundle pacing is generally easier with lumen-less leads, and in case LBBAP with SDL does not give satisfactory results, HBP as backup might not be as easy with SDL. Although, the optimal lead design for LBBAP (and CSP in general) has not yet been determined, several of the features of current SDL may merit incorporation into future dedicated LBBAP lead designs.

## Data Availability Statement

The original contributions presented in the study are included in the article/Supplementary Material, further inquiries can be directed to the corresponding author/s.

## Author Contributions

JD: article concept, drafting, writing, figures, and review. AW: concept and drafting. FV: critical review. J-BL: article concept, writing, and review. All authors contributed to the article and approved the submitted version.

## Conflict of Interest

JD reports speaker fees and honoraria from Medtronic and Biotronik. AW reports speaker and consultancy fees from Biotronik and Boston Scientific. J-BL reports non-significant speaker fees and honoraria for proctoring and teaching activities from Medtronic, Boston Scientific, Abbott, and Biotronik. The remaining author declares that the research was conducted in the absence of any commercial or financial relationships that could be construed as a potential conflict of interest.

## Publisher's Note

All claims expressed in this article are solely those of the authors and do not necessarily represent those of their affiliated organizations, or those of the publisher, the editors and the reviewers. Any product that may be evaluated in this article, or claim that may be made by its manufacturer, is not guaranteed or endorsed by the publisher.

## Supplementary Material

Supplementary Figure 1 **(A)** Currently available delivery sheaths for left bundle branch area pacing. Note that steerable/single curve sheaths are not represented here as the secondary curve appears critical to correctly position the sheath before lead implantation. **(B)** Different types of pacing leads used for left bundle branch area pacing.

Supplementary Movie 1 Preparation of the Solia S lead when used for LBBAP. The helix might be extended in advance. The green stylet-guide is connected to the lead pin and pressed against the silicone coating at the proximal portion of the lead. To pretension the lead, build up the torque on the inner coil is achieved by rotating the green stylet-guide tool 8–10 times clockwise.

Supplementary Movie 2 Screwing of the Solia S lead during LBBAP. The stylet is advanced to the pin of the pacing lead and fast rotations on the outer lead body are applied to achieve a deep septal position.

Supplementary Movie 3 Preparation of the Ingevity pacing lead when used for LBBAP. The helix is extended using the clip-on-tool. A lead end cap is cut and connected to the proximal pin of the pacing lead to avoid helix retraction. The stylet is inserted and kept advanced when screwing.
