# Supplementary figures and images for: A Guide to Left Bundle Branch Area Pacing Using Stylet-Driven Pacing Leads
Source: Front Cardiovasc Med. 2022 Feb 21;9:844152. doi: 10.3389/fcvm.2022.844152 (PMC8899462; doi:10.3389/fcvm.2022.844152)

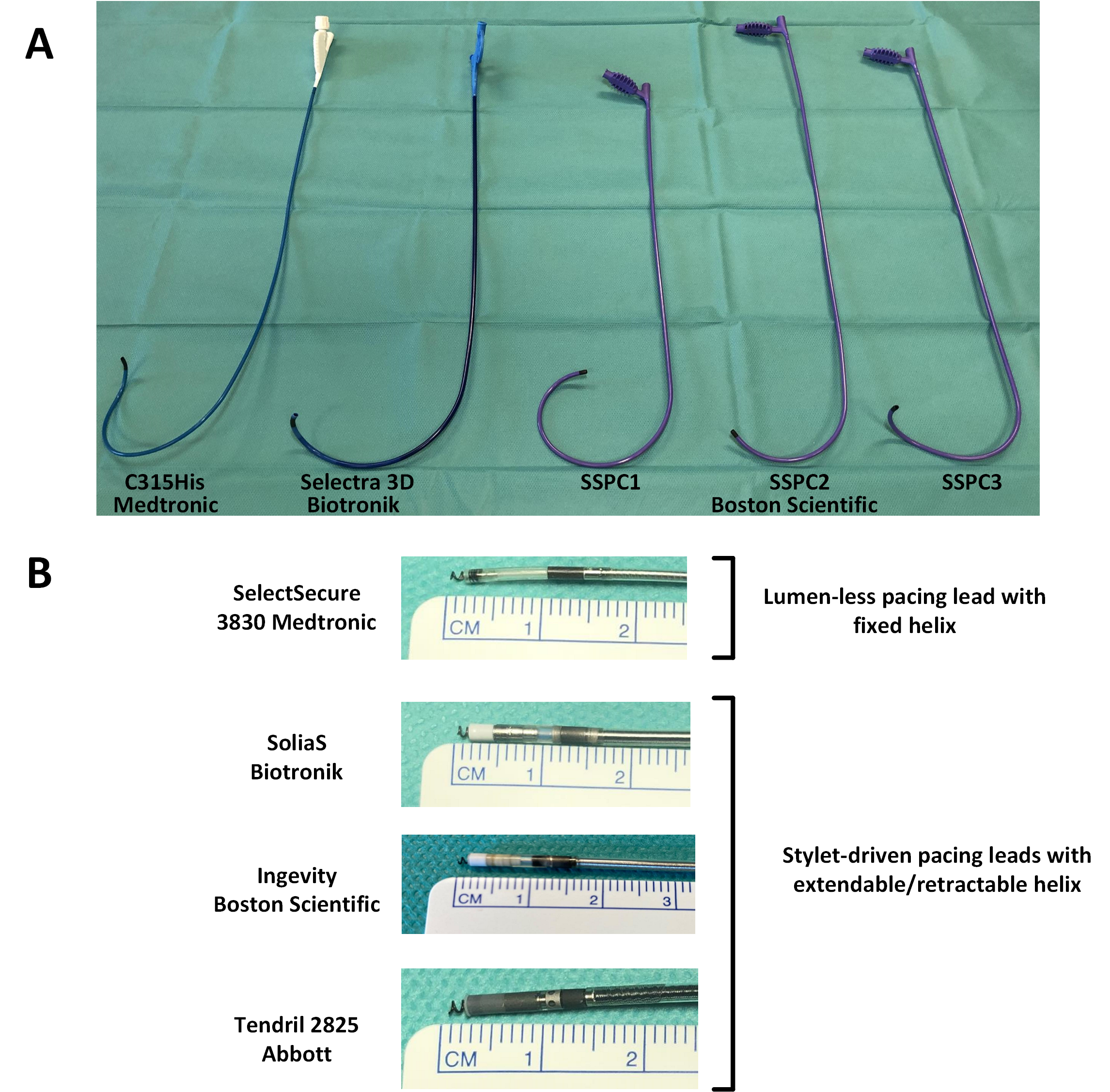

Supplement: Supplementary Figure 1 — (A) Currently available delivery sheaths for left bundle branch area pacing. Note that steerable/single curve sheaths are not represented here as the secondary curve appears critical to correctly position the sheath before lead implantation. (B) Different types of pacing leads used for left bundle branch area pacing. [file Image_1.TIF]
